# Ten-Year Cumulative Incidence of Diabetic Retinopathy. The Beijing Eye Study 2001/2011

**DOI:** 10.1371/journal.pone.0111320

**Published:** 2014-10-27

**Authors:** Jie Xu, Liang Xu, Ya Xing Wang, Qi Sheng You, Jost B. Jonas, Wen Bin Wei

**Affiliations:** 1 Beijing Institute of Ophthalmology, Beijing Tongren Hospital, Capital Medical University, Beijing, China; 2 Department of Ophthalmology, Medical Faculty Mannheim of the Ruprecht-Karls-University, Heidelberg, Germany; 3 Beijing Tongren Eye Center, Beijing Tongren Hospital, Capital Medical University, Beijing, China; Queen’s University Belfast, United Kingdom

## Abstract

**Objective:**

To assess the cumulative 10-year incidence of diabetic retinopathy (DR) and its associated factors in a population living in Greater Beijing.

**Methods:**

The population-based longitudinal Beijing Eye Study, which included 4439 subjects (age in 2001: 40+years) in 2001, was repeated in 2011 with 2695 subjects participating (66.4% of the survivors). The study participants underwent a detailed ophthalmic examination. Fundus photographs were examined for the new development of DR.

**Results:**

After excluding individuals with DR at baseline (n = 87) or no sufficient fundus photographs in 2011 (n = 6), the study included 2602 subjects with a mean age of 64.6±9.7 years (median: 64.0 years; range: 50 to 93 years). In the 10-year period, 109 subjects (39 men) developed new DR with an incidence of 4.2% (95% confidence interval (CI): 3.45,5.03). In multiple logistic regression analysis, incident DR was significantly associated with higher HbA1c value (*P*<0.001; Odds Ratio (OR): 1.73; 95% Confidence Interval (CI): 1.35,2.21), longer duration of diabetes mellitus (*P*<0.001; OR: 1.16; 95% CI: 1.10,1.22), higher serum concentration of creatinine (*P* = 0.02; OR: 1.01; 95% CI: 1.002,1.022), lower educational level (*P* = 0.049; OR: 0.74; 95% CI: 0.55,0.99), higher estimated cerebrospinal fluid pressure (*P* = 0.038; OR: 1.10; 95% CI: 1.01,1.22), and shorter axial length (*P*<0.001; OR: 0.48; 95% CI: 0.33,0.71).

**Conclusions:**

The cumulative 10-year incidence (mean: 4.2%) of DR in a North Chinese population was significantly associated with a higher HbA1c value, longer known duration of diabetes mellitus, higher estimated CSFP and shorter axial length (*P*<0.001). Shorter axial length (or hyperopia) and, potentially, higher CSFP may be additional risk factors to be taken into account when counseling and treating patients with diabetes mellitus.

## Introduction

As one of the leading causes of visual impairment and blindness in the middle-aged population, diabetic retinopathy (DR) has been an increasing threat to quality of life for millions of people worldwide [1]–[3]. Apart from visual morbidity, evidence has been accumulated that the presence of DR indicates an increased risk of systemic vascular diabetic complications including stroke, coronary heart disease and heart failure, and nephropathy [4]. Most of these studies addressing the frequency of DR and its associated factors were designed as cross-sectional investigations, while only few longitudinal studies exist [5]–[17]. These population-based follow-up studies, such as Wisconsin Epidemiologic Study of Diabetic Retinopathy (WESDR), the Blue Mountains Eye Study and the Los Angeles Latino Eye study, were mainly conducted in Western countries. We therefore conducted our study to examine the cumulative incidence of DR in a population living in an urban region and in a developed rural region of Greater Beijing, and simultaneously to explore factors associated with the new development of DR in the study population.

## Methods

The Beijing Eye Study is a population-based prospective cohort study in Greater Beijing that started in 2001 at baseline. The Medical Ethics Committee of Beijing Tongren Hospital approved the study protocol according to the declaration of Helsinki and all study participants gave their written informed consent. The study was divided into an urban part and a rural part. The only eligibility criterion for the study was an age of ≥40 years in the year 2001. There were no exclusion criteria. Out of 5324 eligible individuals, 4439 (83.4%) participated in the baseline study in 2001. In 2006 and 2011, the study was repeated by inviting all participants from the survey of 2001. The details of participants and nonparticipants at baseline and at the 5-year follow-up examination and the examination techniques have been described in detail previously [18], [19]. For the present study, we additionally excluded all individuals who had DR in 2001 or whom sufficient fundus photographs in 2011 were not available.

All study participants underwent an interview with standardized questions on their socioeconomic background, quality of life, psychic depression, physical activity, known major systemic diseases such as arterial hypertension and diabetes mellitus, and quality of vision. The level of education was categorized into the stages of “illiteracy” (no reading ability at all), “semi-illiteracy” (reading ability and knowledge of few Chinese words; no previous school attendance), “primary school education” (previous attendance to a primary school for at least 2 years), “middle school education” (previous attendance to a middle school), and “college or higher education”.

All examinations were carried out in the communities, either in schoolhouses or in community houses. Visual acuity was measured by trained technicians as uncorrected visual acuity (Snellen charts (manufacturer: Precision Vision; IL 60181, USA)) in a distance of 5 m. Near vision was measured in a distance of 25–30 cm (Jaeger charts), uncorrected and then corrected using an addition for near vision. Automatic refractometry (Auto Refractometer AR-610, Nidek Co., Ltd, Tokyo, Japan) was performed if uncorrected distant visual acuity was lower than 1.0. The values obtained by automatic refractometry were verified and refined by subjective refractometry. Intraocular pressure was measured using a non-contact pneumotonometer (CT-60 computerized tonometer, Topcon Ltd., Japan) by an experienced technician. Three measurements were taken, and the mean of the three measurements was taken for further statistical analysis. If the measurements were higher than 25 mm Hg, tonometry was repeated. The pupil was dilated using tropicamide once or twice, until the pupil diameter was at last 6 mm. Using the slit lamp, digital photographs of the cornea and lens were taken. Further examinations were a slit lamp assisted biomicroscopy of the anterior segment of the eye and biometry applying optical low-coherence reflectometry (Lensstar 900 Optical Biometer, Haag-Streit, 3098 Koeniz, Switzerland). Photographs of the macula and optic disc were taken using a fundus camera (Type CR6-45NM, Canon Inc. U.S.A.). For the assessment of age-related maculopathy, the Wisconsin Age-Related Maculopathy Grading system was used as described recently [19].

Fasting blood samples were taken for measurement of blood lipids, glucose and glycosylated hemoglobin HbA1c. Blood pressure was measured. Body height and weight and the circumference of the waist and hip were recorded. For study purposes, we diagnosed diabetes mellitus as any plasma glucose concentration ≥7.0 mmol/L or by a self-reported history of physician diagnosis of diabetes mellitus or by a history of drug treatment for diabetes. Arterial hypertension was defined as systolic blood pressure ≥140 mm Hg and/or diastolic blood pressure ≥90 mm Hg, and/or self-reported current treatment for arterial hypertension.

Using the fundus photographs (non-stereoscopic 45° photograph of the central fundus and of the optic disc (fundus camera type CR6-45NM, Canon Inc., Ōta, Tokyo, Japan)), DR was assessed in a masked manner. The diagnosis for each individual was based on the DR grading of the individuaĺs eye with the highest stage of DR. The grading was performed according to the Early Treatment of Diabetic Retinopathy Study (ETDRS) criteria [20]. The minimum criterion for diagnosis of DR was the presence of at least one microaneurysm. The photographs were assessed by an experienced and trained ophthalmologist (XJ). In case of doubt, the photographs were re-assessed by a panel including several ophthalmologists (JX, LX, QSY, YXW, JBJ, WWB). For study purposes, we defined diabetes mellitus as fasting blood glucose concentration ≥7.0 mmol/L, an HbA1c value ≥6%, by a self-reported history of physician diagnosis of diabetes mellitus, or by a history of drug treatment for diabetes (insulin or oral hypoglycemic agents). Incidence of DR was defined as having no DR in either eye at baseline and having DR of any stage in either eye at the 10-year follow-up.

Cerebrospinal fluid pressure (CSFP) was estimated based on the associations between higher CSFP and younger age, higher body mass index and higher diastolic blood pressure [21]–[23]. The formula used was: “CSFP [mmHg] = 0.44×Body Mass Index [kg/m^2^]+0.16×Diastolic Blood Pressure [mmHg]–0.18×Age [Years] −1.91”. The derivation of the formula has been described in detail previously [21]–[23].

Statistical analysis was performed using a commercially available statistical software package (SPSS for Windows, v. 20.0, IBM- SPSS, Chicago, IL). In a first step, we determined the mean values (presented as mean (SD)) and median values of the main outcome parameters. In a second step, we performed univariate analyses of the associations between the incidence of DR and other systemic parameters and ocular parameters. In a third step, we carried out a multiple logistic regression analyses with the incidence of DR as the dependent parameter and with all those variables as independent parameters that were significantly associated with the incidence of DR in the univariate analyses. We additionally included the concentration of creatinine into the list of independent parameters for the multiple logistic analysis, although this parameter was not significantly with DR incidence in the univariate analysis. Reason was to test a potential association between DR and kidney disease. Out of the list of independent parameters, we then dropped parameters which either showed a high degree of collinearity with other parameters (such as refractive error with axial length) or which were no longer significantly associated with incident DR. Hereby we started with the parameters with the highest *P*-values. Odds ratios (ORs) and 95% confidence intervals were presented. A *P* value<0.05 was considered to indicate statistical significance.

## Results

In 2011, of all 4439 participants who participated in the baseline examination, 2695 individuals were reexamined, whereas 379 participants were dead and 1365 subjects did not agree to be reexamined or had moved away. The response rate was 60.7% of the original cohort, or 66.4% of the survivors. Study participants were significantly younger than nonparticipants (age (in 2001): 54.5±9.7 years versus 57.0±10.8 years; *P*<0.001) and lived more often in the rural region (48.6% versus 42.3%; *P*<0.001). Both groups did not differ in refractive error (–0.35±2.14 diopters versus −0.34±2.18 diopters versus; *P* = 0.23) and gender (men: 42.0% versus 44.0%; *P* = 0.06). Out of the 2695 subjects, we excluded all individuals who had DR in 2001 (87 subjects) or no sufficient fundus photographs in 2011 (6 subjects), so that eventually 2602 subjects were included into the study. The mean age was 64.6±9.7 years (median: 64.0 years; range: 50 to 93 years), the mean refractive error was −0.24±2.21 diopters (median: 0.25 diopters; range: −22.0 to +7.00 diopters), and the mean axial length was 23.2±1.2 mm (median: 23.1 mm; range: 18.96–30.88 mm).

In the 10-year period from 2001 to 2011, 109 subjects (39 men) developed a new DR with an incidence of 4.2% (95% confidence interval (CI), 3.45, 5.03). The mean age of all subjects with incident DR was 64.3±9.3 years (median: 62 years; range: 50–90 years), a mean refractive error of −0.16±1.37 diopters (median: 0.25 diopters; range: −7.38 to +3.13 diopters) and a mean axial length of 22.8±0.8 mm (median: 22.8 mm; range: 20.88–25.10 mm).

In univariate analysis, higher cumulative incidence of DR was significantly associated with the lower level of education (*P*<0.001), higher body mass index (*P* = 0.004), lower body height (*P* = 0.001), longer waist circumference (*P* = 0.005), rural region of habitation (*P*<0.001), longer duration of known diabetes (*P*<0.001), insulin treatment of diabetes (*P* = 0.001), presence of arterial hypertension (*P* = 0.03), higher systolic blood pressure (*P* = 0.005), estimated CSFP (*P* = 0.04), higher serum concentration of glucose (*P*<0.001), a higher HbA1c value (*P<*0.001), and previous cerebrovascular stroke (*P* = 0.03), and with the ocular parameters of shorter axial length (*P*<0.001), smaller corneal curvature radius (*P* = 0.007), thicker subfoveal choroidal thickness (*P* = 0.01) (Table 1). Incidence of DR was not significantly associated with age (*P* = 0.81), gender (p = 0.18), ever smoking (*P* = 0.23) and smoking package years (*P* = 0.42), quantity of alcohol consumption (*P* = 0.42), serum concentrations of high-density lipoproteins (*P* = 0.11), low-density lipoproteins (*P* = 0.66) and triglycerides (*P* = 0.56), and with the ocular parameters of intraocular pressure (p = 0.67), optic disc size (*P* = 0.44), age-related macular degeneration (*P* = 0.44) and glaucoma (*P* = 0.54).

**Table 1:** Associations (Univariate Analysis) between Ten-Year Incidence of Diabetic Retinopathy in the Beijing Eye Study.

Parameter	*P*-Value	Odds Ratio	95% Confidence Interval
General Parameters			
Age (Years)	0.81	1.00	0.98, 1.02
Gender	0.18	1.32	0.88, 1.96
**Level of Education**	**<0.001**	**0.75**	**0.64, 0.88**
**Rural/Urban Region of Habitation**	**<0.001**	**0.44**	**0.29, 0.66**
**Body Mass Related Parameters**			
**Body Mass Index (kg/m^2^)**	**0.004**	**1.07**	**1.02, 1.12**
**Body Height (cm)**	**0.001**	**0.96**	**0.94, 0.98**
Body Weight (kg)	0.63	1.00	0.99, 1.02
**Waist Circumference (cm)**	**0.005**	**1.03**	**1.01, 1.05**
Diet, Smoking, Alcohol			
Fish-Rich Diet	0.66	1.01	0.96, 1.07
Vegetable-Rich Diet	0.07	1.09	0.99, 1.19
Meat-Rich Diet	0.59	0.86	0.50, 1.48
Smoking, Ever	0.23	1.28	0.85, 1.91
Smoking Package Years	0.42	1.00	0.99, 1.01
Alcohol Consumption Quantity	0.42	1.06	0.92, 1.23
Alcohol Consumption Frequency	0.43	0.95	0.85, 1.07
Diabetes Mellitus-Related Parameters			
**Previous Cerebrovascular Stroke**	**0.03**	**1.94**	**1.06, 3.55**
**Known Duration of Diabetes Mellitus (Years)**	**<0.001**	**1.12**	**1.09, 1.15**
**Diabetes Treated with Insulin**	**0.001**	**3.12**	**1.56, 6.25**
Diabetes Treated with Oral Anti-Diabetic Medication	0.38	1.76	0.50, 6.14
**Diabetes Treatment None/oral Medication/Insulin**	**0.002**	**2.57**	**1.42, 4.64**
**Arterial Hypertension**	**0.03**	**1.57**	**1.06, 2.34**
**Systolic Blood Pressure (mmHg)**	**0.005**	**1.012**	**1.004, 1.021**
Diastolic Blood pressure (mm Hg)	0.35	1.007	0.992, 1.023
**Estimated Cerebrospinal Fluid Pressure (mmHg)**	**0.04**	**1.06**	**1.002, 1.11**
Biochemical Blood Examinations			
**Serum Concentration of Glucose (mmol/L)**	**<0.001**	**1.47**	**1.34, 1.61**
Serum Concentration of Triglycerides (mmol/L)	0.56	1.02	0.97, 1.07
Serum Concentration of High-Density Lipoproteins (mmol/L)	0.11	0.65	0.38, 1.10
Serum Concentration of Low-Density Lipoproteins (mmol/L)	0.66	1.05	0.84, 1.33
**Glycosylated Hemoglobin HbA1c (%)**	**<0.001**	**1.97**	**1.69, 2.29**
C-Reactive Protein	0.26	1.02	0.98, 1.06
Creatinine	0.39	1.01	0.99, 1.02
Ocular Parameters			
**Axial Length (mm)**	**<0.001**	**0.62**	**0.50, 0.76**
Refractive Error (Diopters)	0.05	1.12	1.00, 1.26
Anterior Chamber Depth	0.11	1.33	0.94, 1.88
Lens Thickness	0.53	1.23	0.65, 2.30
Central Corneal Thickness (µm)	0.49	1.00	0.99, 1.00
**Corneal Curvature Radius (mm)**	**0.007**	**0.34**	**0.15, 0.75**
**Subfoveal Choroidal Thickness (µm)**	**0.01**	**1.002**	**1.001, 1.004**
Intraocular Pressure (mmHg)	0.67	1.02	0.95, 1.09
Retinal Nerve Fiber Layer Thickness (µm)	0.50	1.01	0.99, 1.02
Optic Disc Area (mm^2^)	0.44	0.85	0.57, 1.28
Age-Related Macular Degeneration (Early Stage)	0.44	1.28	0.68, 2.44
Glaucoma	0.54	1.26	0.60, 2.64
Open-Angle Glaucoma	0.80	1.12	0.45, 2.82
Angle-Closure Glaucoma	0.43	1.62	0.49, 5.30

In the first step, the binary regression analysis included the incidence of DR as dependent variable and as independent variables all those parameters which were significantly associated with DR incidence in the univariate analysis. We first dropped parameters (such as refractive error, body height and waist circumference) which showed a high degree of collinearity with other parameters. We then dropped parameters which were no longer significantly associated with incident DR starting with the parameters with the highest *P*-values. The final model showed that incident DR remained to be significantly associated with higher HbA1c value (*P*<0.001), longer duration of diabetes mellitus (*P*<0.001), higher serum concentration of creatinine (*P* = 0.02), lower educational level (*P* = 0.049), higher estimated CSFP (*P* = 0.038), and shorter axial length (*P*<0.001) (Table 2). If the parameter of previous cerebrovascular strokes was added into the list of independent variables, higher DR incidence was marginally associated with a higher prevalence of previous stroke (*P* = 0.58; OR: 2.55; 95% CI: 0.97, 6.73). The results did not markedly change if the list if independent variables additionally include presence of hyperlipidemia (*P* = 0.16; OR: 1.06; 95% CI: 0.98, 1.15), serum concentration of high-density lipoproteins (*P* = 0.54; OR: 1.34; 95% CI: 0.53, 3.39), serum concentration of low-density lipoproteins (*P* = 0.74; OR: 1.06; 95% CI: 0.74, 1.53), serum concentration of triglycerides (*P* = 0.94; OR: 0.99; 95% CI: 0.81, 1.22), or smoking (*P* = 0.25; OR: 1.49; 95% CI: 0.75, 2.98).

**Table 2:** Associations (Multivariate Analysis) between Ten-Year Incidence of Diabetic Retinopathy in the Beijing Eye Study.

Parameter	*P*-Value	OddsRatio	95% Confidence Interval
Glycosylated Hemoglobin HbA1c (%)	<0.001	1.73	1.35, 2.21
Known Duration of Diabetes Mellitus (Years)	<0.001	1.16	1.10, 1.22
Estimated Cerebrospinal Fluid Pressure (mmHg)	0.038	1.10	1.01, 1.21
Serum Concentration of Creatinine (mmol/L)	0.02	1.01	1.002, 1.022
Level of Education	0.049	0.74	0.55, 0.99
Axial Length (mm)	<0.001	0.48	0.33, 0.71

## Discussion

In our longitudinal population-based study, the cumulative 10-year incidence of DR was 4.2% (95% CI: 3.45, 5.03), higher DR incidence was significantly associated with higher HbA1c value (*P*<0.001), longer duration of diabetes mellitus (*P*<0.001), higher estimated CSFP (*P* = 0.038), and shorter axial length (*P*<0.001). There were marginal associations between a higher incidence of DR and higher serum concentration of creatinine (*P* = 0.02) and a lower level of education (*P* = 0.049). Incidence of DR was not significantly associated with hyperlipidemia or smoking. The figure of the 10-year incidence of DR in our study population from North China agrees with studies from other ethnic groups and countries [10], [24]. Our population-based study also agrees with previous hospital-based and population-based investigations on associations between incident DR and longer known duration of diabetes mellitus and the concentration of HbA1c [17], [25]–[27].

On a longitudinal basis, our study reveals the new finding on an association between the incidence of DR and shorter ocular axial length. It is in agreement with previous cross-sectional studies in which individuals with shorter axial length as compared to subjects with longer eyes showed a higher prevalence of DR [28]–[30]. In the cross-sectional, clinic-based study by Man and colleagues on patients with diabetes aged 18 years or more, eyes with longer axial length were less likely to have mild (OR: 0.58; 95% CI: 0.41, 0.83; per mm increase in axial length), moderate (OR: 0.73; 95% CI: 0.60, 0.88), and severe DR (OR: 0.67; 95% CI: 0.53,0.85; P = 0.01) [28]. In the population-based, cross-sectional Singapore Malay Eye Study, eyes with longer axial length were less likely to have any DR (OR: 0.86; 95% CI: 0.75,0.99; per 1-mm increase), moderate DR (OR, 0.80; 95% CI, 0.62–1.05), and vision-threatening DR (OR, 0.63; 95% CI, 0.40–0.99) (29). Pierro et al. reported that axial length was shorter in diabetic patients than in non-diabetic subjects, and that within the diabetic group, patients with retinopathy had shorter axial lengths than did patients without retinopathy in multivariate analysis [30]. The association between shorter axial length and higher prevalence and incidence of DR is parallel to the association between shorter axial length and higher prevalence and incidence of age-related macular degeneration [31]. Since the intravitreal concentration of cytokines such as vascular endothelial growth factor increase with shorter axial length, one may infer that the smaller intraocular volume in eyes with shorter axial length in combination with a firmer vitreous body may lead to higher intraocular concentrations of cytokines which are associated with DR [32]. Interestingly, the association between higher incidence of DR and shorter axial length remained valid after adjusting for educational level and other parameters (Table 2). Shorter axial length or hyperopia has been reported to be associated with a lower socioeconomic background, so that by not including the socioeconomic background into the multivariate model, a bias might be introduced [33], [34].

Incidence DR was additionally associated with a higher estimated CSFP in our study population. After adjusting for the level of HbA1c, known duration of diabetes, serum concentration of creatinine, educational level and axial length, the 10-year incidence of DR increased by a factor of 1.10 for an increase in estimated CSFP by one mm Hg (Table 2). It is in agreement with results of recent studies in which larger retinal vein diameters were significantly associated with higher estimated CSFP (*P* = 0.001), in which a higher 10-year incidence of retinal vein occlusions was correlated with higher estimated CSFP, and in which the prevalence and severity of DR was significantly associated with higher CSFP (*P* = 0.006) [35], [36]. Correspondingly, the Wisconsin Epidemiologic Study of Diabetic Retinopathy revealed that independently of DR severity level, glycemic control and other factors, widening of retinal venular caliber but not arteriolar diameter was associated with subsequent incidence and progression of DR [8]. The reason for these associations may be the anatomy of the retinal venous blood system which drains through the central retinal vein through the optic nerve and the orbital cerebrospinal fluid space via the superior ophthalmic vein into the intracranial venous system. One may infer that the blood pressure in the central retinal vein inside of the eye is at least as high as the CSFP. An experimental study in monkeys accordingly showed that the central retinal vein pressure and CSFP were directly correlated with each other, in the normal state and in the situation of an elevated CSFP [37]. An elevated retinal vein pressure in patients with higher CSFP will be associated with a higher retinal capillary blood pressure potentially explaining the increased incidence and prevalence of retinal hemorrhages, edema and lipid exudates as part of DR. As recently suggested by Stodtmeister and colleagues, an elevated retinal venous pressure due to an increased CSFP may additionally decrease the ocular perfusion pressure defined as the difference between the retinal arterial blood pressure and the retinal venous blood pressure [38]. A decrease in the ocular perfusion pressure leads to an increase in the risk for ischemic retinopathies such as DR. If the association between higher CSFP and incidence of DR is further clarified in future studies, one may address the question whether lowering of CSFP by drugs such as systemic carbonic anhydrase inhibitors may have a therapeutically positive effect on the development of DR. The association between higher estimated CSFP and DR may also explain the dilatation of retinal veins and their increased tortuosity as hallmarks of DR. An increased arterial blood pressure alone may not explain why on the venous side of the vascular bed the vessels get wider.

In our study, lower level of education was another marginally significant risk factor for an increased incidence of DR in the multivariate analysis (Table 2). Correspondingly, rural region of habitation with a lower mean educational level as compared to urban regions was associated with a higher incidence of DR in the univariate analysis (Table 1). It may show the importance of the socioeconomic background in terms of lifestyle, understanding of the importance of a therapy of diabetes and the financial possibilities to do so. Designers of future studies on DR and on diabetes mellitus in general may consider including the educational level or other parameters of the socioeconomic background into the study designs.

Interestingly, incidence of DR was not significantly associated with age (*P* = 0.81). A similar finding was obtained for the very rural population of the recent Central India Eye and Medical Study in which the association between the DR prevalence and age showed an inverse U-shape with an increase from the age group of 30–39 years with a prevalence of 0.00% to the age group of 50–59 years with a prevalence of 0.63±0.28% and the age group of 60–69 years with a prevalence of 0.71±0.29%, and a decrease in DR prevalence thereafter (70–79 years: 0.27±0.27%; 80+ years: 0.00%). One may postulate that in Central India as in some parts of Greater Beijing, a shortened life expectancy of patients with diabetes may have prevented a clearly positive association between increasing prevalence or incidence of DR and older age [39].

Potential limitations of our study should be mentioned. First, as in any population-based study, selection bias could have accentuated some estimates and masked others. The overall participation rate in our survey was 60.7% of the original cohort, or 66.4% of the survivors, so it is possible that nonparticipation may have influenced the results of our study. Compared with other 10-year follow-up studies in ophthalmic epidemiology, the response rate in our study was lower than that in the Blue Mountains Eye Study (75.6% of survivors) and the Beaver Dam Eye Study (82.9% of survivors). The reason for the lower follow-up response in the current study is the presumably higher mobility of the population in Greater Beijing compared with the mobility of the populations from the Blue Mountains Eye Study and Beaver Dam Eye Study. Because of intensive land development activities in the rural region and the urban regions of the Beijing Eye Study, a substantial number of inhabitants moved away during the follow-up period. A major reason to move was the planning and construction of a new airport in the vicinity of some of the villages. Because the reason to move was independent of the general health condition but depended on the location of the houses, it may not have introduced a major bias into the study. Second, the nonparticipants were not fully comparable to the study participants, so that the non-participation may have influenced the results of the study. Third, only two non-stereoscopic fundus photographs (taken of the central fundus and of the optic nerve head) were used to detect diabetic changes in the retina in our study, while the ETDRS criteria use 7-field stereo images. This difference may have led to an underestimation in the incidence of DR in our study.

In conclusion, the cumulative 10-year incidence of DR with a mean of 4.2% in the adult population of Greater Beijing was significantly associated with a higher HbA1c value, longer known duration of diabetes mellitus, higher estimated CSFP and shorter axial length. There were marginally significant associations with a higher serum concentration of creatinine and a lower educational level. Incidence of DR not significantly associated with hyperlipidemia or smoking. The association with shorter axial length and higher estimated CSFP may warrant further investigation.
